# Vanadium-Oxide-Based Thin Films with Ultra-High Thermo-Optic Coefficients at 1550 nm and 2000 nm Wavelengths

**DOI:** 10.3390/ma13082002

**Published:** 2020-04-24

**Authors:** Mohamed Abdel-Rahman, Esam Bahidra, Ahmed Fauzi Abas

**Affiliations:** 1 Department of Electrical Engineering, College of Engineering, King Saud University, Riyadh 11421, Saudi Arabia; esambahidra@gmail.com (E.B.); aabas@ksu.edu.sa (A.F.A.); 2 KACST-TIC in Radio Frequency and Photonics for the e-Society (RFTONICS), College of Engineering, King Saud University, Riyadh 11421, Saudi Arabia

**Keywords:** vanadium sesquioxide, thin film, thermo-optic coefficient, infrared ellipsometry, optical constants

## Abstract

In this paper, the temperature-dependent dielectric properties of vanadium-sesquioxide-based thin films are studied to assess their suitability for thermally tunable filters at optical communication wavelengths. Spectroscopic ellipsometry is utilized to measure the optical constants of vanadium oxide thin films at temperatures ranging from 25 °C to 65 °C. High thermo-optic coefficients (*dn*/*dT*s) were observed. The highest *dn*/*dT*s, measured at approximately 40 °C, were −8.4 × 10^−3^/°C and −1.05 × 10^−2^/°C at 1550 nm and 2000 nm, respectively.

## 1. Introduction

Tunable optical filters are crucial components in Wavelength Division Multiplexing (WDM) transmission systems, optical amplifier development, noise filtering and many more. Tunable optical filters provide a means for isolating a desired wavelength from noises, and have become enabling technologies for many optical devices and systems. Tunable optical filters have been realized based on several technologies since the last decade [1,2,3,4]. One of those technologies is thermo-optic tuning, which evolved rapidly, introducing an alternative method to develop tunable optical filters [2,3,4]. In this technology, the key performance parameter is the thermo-optic coefficient (*dn*/*dT*), which is a parameter that quantifies the change in a material’s refractive index due to a temperature change. Realizing a thin film material with a high *dn*/*dT* at the optical communication wavelengths is an enabler for high performance thin-film-based tunable optical filters [4]. 

Vanadium-sesquioxide-based thin films prepared by the multilayer synthesis technique have shown high *dn*/*dT*s (up to 6.2 × 10^−3^/°C at room temperature) at midwave and longwave (3000 to 12,000 nm) infrared wavelengths [5]. This fact encouraged studying the properties of vanadium oxide thin films developed in the literature [5] at optical communication wavelengths, 1550 nm and 2000 nm, for potential use in thermally tunable filters. In this work, we utilize infrared spectroscopic ellipsometry for studying the temperature-dependent ellipsometric parameters of vanadium-sesquioxide-based thin films. The Drude–Lorentz dispersion model is then used to extract the temperature-dependent optical properties of the films. The temperature-dependent optical properties are analyzed, revealing high *dn*/*dT*s at 1550 nm and 2000 nm.

## 2. Film Synthesis and Ellipsometric Data Acquisition

A vanadium-sesquioxide-based thin film was prepared using the sputter deposition method. The 95 nm-thick vanadium oxide film was deposited on a silicon substrate (380 µm-thick) with a 300 nm-thick layer of thermally grown silicon dioxide on top. The film was prepared by sputter depositing 19 alternating layers of vanadium sesquioxide (V_2_O_3_) and vanadium (V). The thickness of each of the V_2_O_3_ and V layers was 5 nm. The deposition was followed by an annealing step at 300 °C in an O_2_ atmosphere for 30 min. The alternating multilayer deposition followed by annealing caused the diffusion of O_2_ molecules from the V_2_O_3_ oxygen-rich layers to the V layers. This technique yielded mixed phase vanadium oxide films [6] with electrical and optical properties that were tunable, in accordance with the synthesis process parameters [5,6]. Further details of the vanadium oxide film synthesis process can be found in the literature [5]

**Figure 1:**
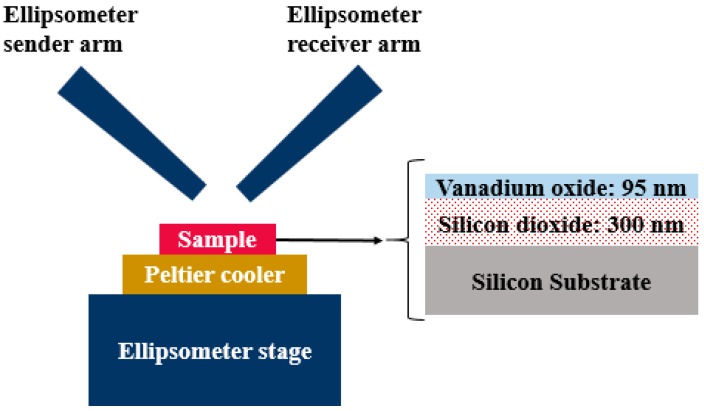
A schematic diagram of the ellipsometer setup used to measure the psi (*Ψ*) and delta (*Δ*) parameters of the vanadium oxide/silicon dioxide/silicon substrate structure.

Following the vanadium oxide film synthesis, the ellipsometric parameters, psi (*Ψ*) and delta (*Δ*), of the vanadium oxide/silicon dioxide/silicon substrate structure were measured using a Sendira infrared spectroscopic ellipsometer, where *Ψ* and *Δ* are the amplitude ratio and the phase shift between the perpendicular and parallel components, respectively, of the wave reflected from the sample. For this purpose, the silicon substrate with the deposited vanadium oxide thin film was placed on the spectroscopic ellipsometer’s sample holder. A Peltier cooler was used to vary the temperature of the sample from 25 °C to 65 °C in steps of 10 °C. A schematic diagram of the measurement setup is shown in Figure 1. The *Ψ* and *Δ* parameters were acquired at 50° and 60° angles of incidence over a spectral range of 1540 nm to 2800 nm (0.8051 eV to 0.442 eV) in 1 nm steps.

## 3. Modeling Ellipsometric Data

The spectral dependencies of the *Ψ* and *Δ* ellipsometric data were modeled using SpectraRay ellipsometric analysis software [7]. The optical constants for the silicon substrate and the silicon dioxide layer were imported from the software’s materials library. The optical constants of the vanadium oxide layer were analyzed by modeling the thin film layer using the Drude–Lorentz dispersion model, given by the following [7]:

(1) $$\varepsilon \left(\omega \right)=\varepsilon_{\infty }+\underset{j}{\sum }\frac{f_{j}\omega_{oj}^{2}}{\omega_{oj}^{2}-\omega^{2}-i\Gamma_{j}\omega }-\frac{\omega_{p}^{2}}{\omega^{2}+i\omega_{\tau }\omega }$$

where *ε_∞_* is the dielectric constant and ε∞ is the high-frequency dielectric constant, and *ω_p_* and *ω_τ_* are the plasma frequency and damping frequency, respectively. Furthermore, *ω_oj_*, *f_j_* and Γ_*j*_ are the resonance frequency, strength factor and damping constant for the j-th oscillator, respectively. 

The *Ψ* and *Δ* ellipsometric data for the vanadium oxide thin film were measured at 5 different temperatures, and so the vanadium oxide thin film was modeled separately at each temperature. In all of the models, the high-frequency dielectric constant (ε∞) was taken to be 1, and the dielectric function was fitted using 3 Lorentz harmonic oscillators. Table 1 summarizes the Lorentz-oscillator model fitting parameters for the vanadium oxide thin film at 5 different temperatures. Table 2 summarizes the Drude-oscillator model fitting parameters for the vanadium oxide thin film at 5 different temperatures. 

**Figure 2:**
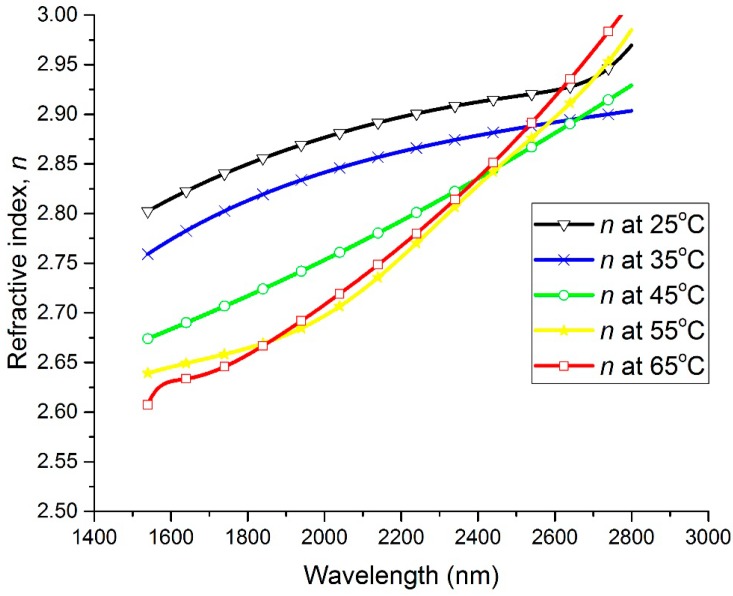
Refractive index (*n*) versus wavelength at temperatures ranging from 25 °C to 65 °C.

**Figure 3:**
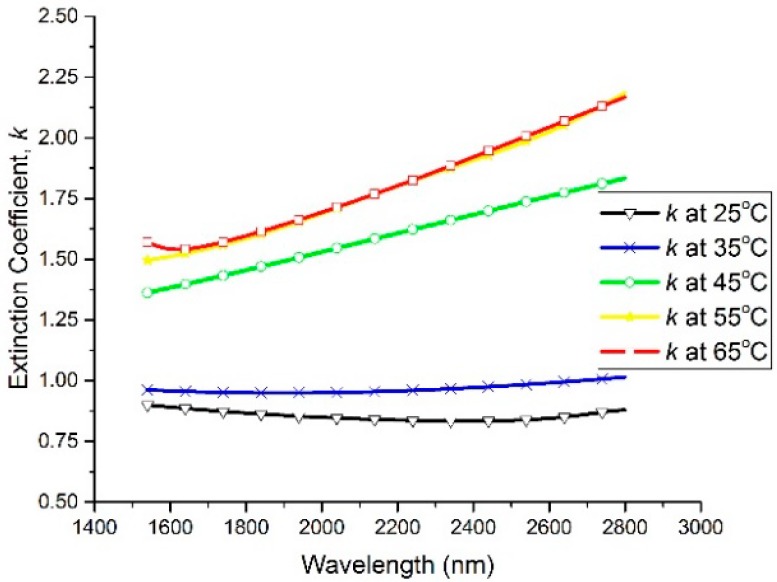
Extinction coefficient (*k*) versus wavelength at temperatures ranging from 25 °C to 65 °C.

**Figure 4:**
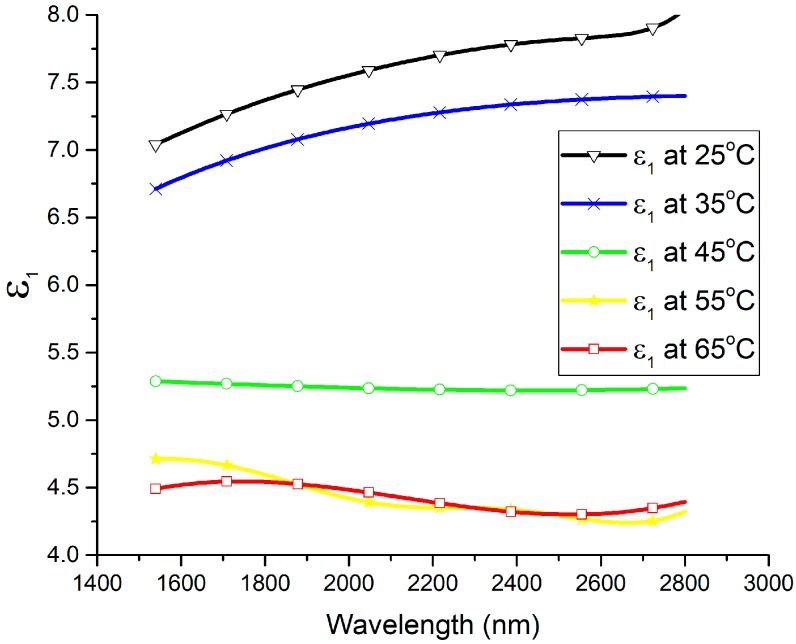
Real part of the material’s permittivity (*ε_1_*) versus wavelength at temperatures ranging from 25 °C to 65 °C.

**Figure 5:**
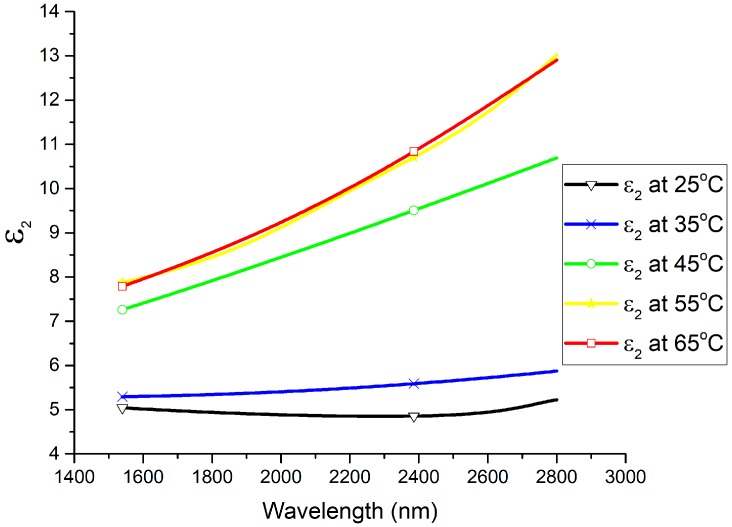
Imaginary part of the material’s permittivity (*ε_2_*) versus wavelength at temperatures ranging from 25 °C to 65 °C.

The spectral dependencies of the *n* and *k* optical constants are plotted at different temperatures in Figure 2 and Figure 3, respectively. In the wavelength range of 1540 nm to 2200 nm, the refractive index (*n*) exhibits a large decrease with the increase in the temperature of the thin film, while the extinction coefficient (*k*) shows a large increase with the increase in the thin film’s temperature. It can be seen that *n* decreased from 2.8 to 2.62 at 1550 nm and from 2.88 to 2.71 at 2000 nm by increasing the temperature from 25 °C to 65 °C, whereas *k* increased from 0.9 to 1.56 at 1550 nm and from 0.85 to 1.7 at 2000 nm by increasing the temperature from 25 °C to 65 °C. Furthermore, the spectral dependencies of the complex permittivity components ε_1_ (ε_1_ = *n*^2^ − *k*^2^) and ε_2_ (ε_2_ = 2*nk*) are plotted at different temperatures in Figure 4 and Figure 5, respectively. The plots indicate that the real part of the material’s permittivity (ε_1_) decreased as the temperature increased, and the imaginary part of the material’s permittivity (ε_2_) increased with the increase in temperature. We compared the temperature-dependent behavior of the measured permittivities to that of the pure V_2_O_3_ thin films developed in the literature [8] by observing the approximate values of ε_1_ and ε_2_ at 0.7 eV (corresponding to a wavelength between 1.55 µm and 2 µm [8]. The measured ε_1_ of the pure V_2_O_3_ thin films in the literature [8] decreased from ≈5 to ≈0.5 as the temperature increased from 100 K to 200 K. In addition, the measured ε_2_ of the pure V_2_O_3_ thin films in the literature [8] increased from ≈1 to ≈7 as the temperature increased from 100 K to 200 K. The behavior of the vanadium oxide film studied in this paper followed the same trend, i.e., a decrease in ε_1_ with an increase in the temperature, and an increase in ε_2_ with an increase in the temperature. This confirms that the vanadium oxide film synthesized in this work contained V_2_O_3_.

## 4. Analysis and Discussion

**Figure 6:**
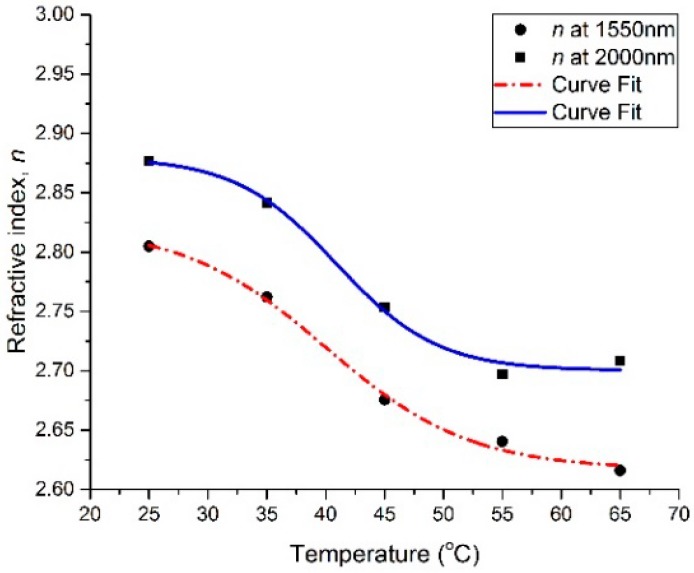
Refractive index (*n*) versus temperature at 1550 nm and 2000 nm wavelengths.

**Figure 7:**
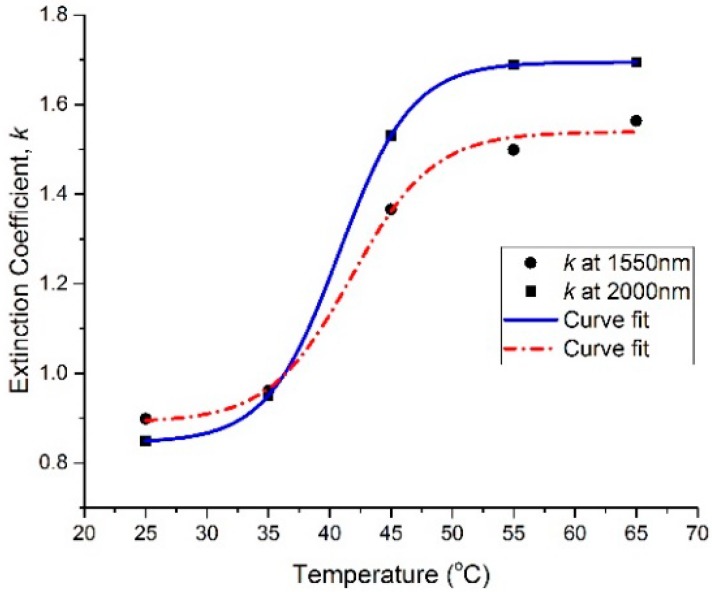
Extinction coefficient (*k*) versus temperature at 1550 nm and 2000 nm wavelengths.

The results were further analyzed by plotting the *n* and *k* versus temperature at 1550 nm and 2000 nm in Figure 6 and Figure 7, respectively. The plotted data points in Figure 6 and Figure 7 were fitted using a sigmoidal curve fit. Both *n* and *k* show a small change before and after a transitional temperature range, from approximately 35 °C to 55 °C, whereas a large change in *n* and *k* can be observed. The observed sharp transition can be due to a change in the film’s behavior from being semiconducting to lossy-metallic; this interpretation is supported by the measured temperature-dependent resistive behavior in the literature [5]. Furthermore, the results in Figure 7 indicate that the developed thin film was lossy with a high extinction coefficient, and had a corresponding high absorption coefficient throughout the whole temperature range.

**Figure 8:**
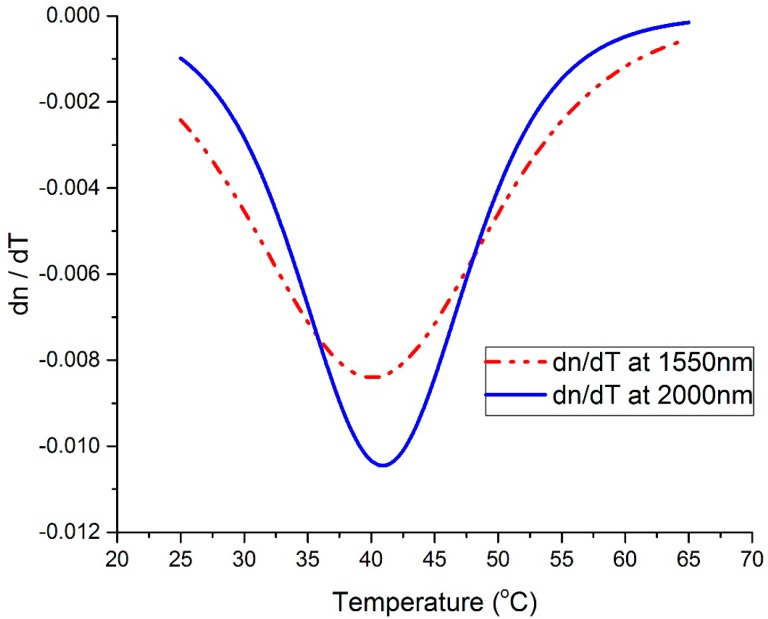
*dn*/*dT* versus temperature at 1550 nm and 2000 nm wavelengths.

The results in Figure 6 were further analyzed to quantify the thermo-optic coefficient (*dn*/*dT*) of the vanadium oxide thin film under analysis. This was done by taking the first derivative of the curve in Figure 6. In Figure 8, the *dn*/*dT* is plotted versus temperature at 1550 nm and 2000 nm. From Figure 8, it can be seen that the vanadium oxide film had a high *dn*/*dT* at room temperature, −3.16 × 10^−3^/°C at 1550 nm and −1.52 × 10^−3^/°C at 2000 nm. The highest sensitivity at 1550 nm occurred at 39.9 °C, where the *dn*/*dT* reached −8.4 × 10^−2^/°C, and the highest *dn*/*dT* at 2000 nm was −1.04 × 10^−2^/°C, measured at 40.93 °C. The achieved *dn*/*dT*s in this work are considerably high compared with the *dn*/*dT* of the amorphous silicon films that were developed in in the work of [4] for the same application; the *dn*/*dT*s of the amorphous silicon films were 3.6 × 10^−4^/°C. In addition, the achieved *dn*/*dT*s in this work are also high compared with the *dn*/*dT*s of the zinc oxide thin films (−2.7 × 10^−4^/°C) [9], tantalum oxide thin films (4.76 × 10^−5^/°C) [10], aluminum oxide thin films (4.92 × 10^−5^/°C) [10] and titanium oxide thin films (−2.31 × 10^−5^/°C) [10], all of which were developed for other optoelectronic applications. Vanadium oxide is known to be a thermometer material that follows Arrhenius behavior, with a high temperature coefficient of resistance. A high temperature coefficient of resistance means that the activation energy of the material is high, and the conductivity of the material is highly sensitive to temperature. Accordingly, when the temperature of the vanadium oxide material changes, it will have a big change in the amount of free carriers. Carriers are known to interact with incoming light, causing a change in the optical properties. Furthermore, the developed vanadium oxide thin films exhibited a quasi-phase transition behavior in the measurement temperature range, deduced from the resistance versus temperature behavior [5], all of which may yield a high *dn*/*dT*.

The realized high *dn*/*dT*s indicate the possibility of using the synthesized thin film in tunable filters at 1550 nm and 2000 nm. The developed vanadium oxide thin film can compose or partially compose an active cavity layer in a thin-film-based Fabry–Pérot filter configuration. The filter can be set to operate where the extinction coefficient change is the lowest or nearly zero, at approximately 55 °C. The developed thin film may be best employed in tapped tunable filters where insertion loss is not critical.

## 5. Conclusions

In this paper, the temperature-dependent dielectric properties of vanadium oxide thin films were studied using infrared spectroscopic ellipsometry. The vanadium oxide films under study were synthesized by alternately sputter depositing ultra-thin films of V_2_O_3_ and V, and further annealing the cascaded thin film structure in the O_2_ atmosphere. The ellipsometric measurements were analyzed by modeling the vanadium oxide films using the Drude–Lorentz oscillator model, and the temperature dependent dielectric properties were extracted. The refractive index (*n*) varied from 2.8 to 2.62 at 1550 nm and varied from 2.88 to 2.71 at 2000 nm, while *k* varied from 0.9 to 1.56 at 1550 nm and varied from 0.85 to 1.7 at 2000 nm, by increasing the temperature from 25 °C to 65 °C. The studied vanadium oxide thin films showed high thermo-optic coefficients at room temperature and up to 65 °C. At ≈40 °C, the thermo-optic coefficients reached −8.4 × 10^−2^/°C at 1550 nm and −1.04 × 10^−2^/°C at 2000 nm. The results encourage the exploration of the developed vanadium oxide thin films in thermally tunable Fabry–Pérot filters or in other optical communication devices requiring temperature tunability.

## Figures and Tables

**Table 1:** Summary of the Lorentz-oscillator model fitting parameters.

Temperature	Oscillator Number	*ω_o_* (1/cm)	*f* (1/cm)	Γ (1/cm)
25 °C	1st	386,953.06	277,272.31	219,592.61
	2nd	3510.58	887.41	473.66
	3rd	40,051.91	101,286.24	140,403.64
35 °C	1st	49,062.24	127,980.05	214,428.97
	2nd	8084.86	26,587.91	168,517.97
	3rd	3715.14	38,778.30	359,784.53
45 °C	1st	304,755.16	203,098.52	67,040.95
	2nd	6270.27	41,878.19	83,629.83
	3rd	127,295.33	211,269.44	399,999.47
55 °C	1st	4443.55	3110.98	2020.83
	2nd	2988.94	6828.43	1673.94
	3rd	37,461.18	121,078.52	212,566.64
65 °C	1st	1339.63	3648.94	456.64
	2nd	2297.91	8245.61	2153.14
	3rd	31,002.16	114,284.49	219,101.48

**Table 2:** Summary of the Drude-oscillator model fitting parameters.

Temperature	*ω_p_* (1/cm)	*ω_τ_* (1/cm)
25 °C	4404.65	4990.92
35 °C	4083.51	1690.66
45 °C	5408.59	2742.11
55 °C	6325.33	421
65 °C	1339.63	0
